# Effects of Camphor

**Published:** 1844-10

**Authors:** 


*Effects of Camphor.*—M. Raspail in his valuable lectures on the physiology of health and disease, says on this subject—During the last five years, I have been in the habit of smoking and inhaling camphor, under the form of a cigar, both day and night; I have also placed every night, under my bolster, a certain quantity of purified camphor. My nights, instead of being agitated, have been passed in a calm and uninterrupted sleep. Indifferent dreams, recalling but the ordinary scenes of life, have succeeded to terrific nightmares, which used to torment me, almost every night, for at least a quarter of an hour. Whenever I awake, I chew from fifteen to twenty *centigrammes* (3 to 4 grains) at least of camphor, which I afterwards swallow, along with a small quantity of water; this sometimes amounts, in the course of the night, to as much as sixty *centigrmmes* (12 grains) of camphor, which I have accustomed myself to swallowing; in the day-time, I often take a dose of similar strength; as an hygienic precaution, I also use frictions of camphorated spirits, when rising or going to bed, and whenever I perceive the least lassitude of spirit, or the slightest exhaustion of body. And with this inflammatory treatment according to the Brownian, the Rasorian physiological doctrines, I never was better in my life, nor, in fact, so well for a long time together; I have entered on a new kind of existence; I have, so to speak, shed the old skin of disease; I have grown young again in physical and moral strength; I am more disposed to labor, and am less inconvenienced than ever by it. I therefore consider myself justified in recommending others to partake of the benefits derived from this long and conclusive trial. My own family, as well as numerous patients, can bear out my testimony as to the immense advantages they have derived, from this medicinal agent. I should add that constipation is, generally speaking, produced by medicines of this class; this constitutes the reverse to their good effects, to the activity which they excite in the digestive organs, and the appetite to which they give rise.—*Braithwaite's Retrospect—from Med. Times.*

